# Acute type A aortic dissection involving the iliac and left renal arteries, misdiagnosed as myocardial infarction

**DOI:** 10.5830/CVJA-2017-042

**Published:** 2018

**Authors:** Nkemtendong Tolefac Paul, Dzudie Anastase, Mouliom Sidick, Hentchoya Romuald, Aminde Leopold, H Abanda Martin, Mve Mvondo Charles, D Wanko Vanina, N Luma Henry

**Affiliations:** Faculty of Medicine and Biomedical Sciences, University of Yaoundé I, Yaoundé, Cameroon; Cardiac Intensive Care Unit, Douala General Hospital, Douala Cameroon; Cardiac Intensive Care Unit, Douala General Hospital, Douala Cameroon; Cardiac Intensive Care Unit, Douala General Hospital, Douala Cameroon; Faculty of Medicine and Biomedical Sciences, University of Queensland, Australia; Cardiavascular Centre, Douala, Cameroon; Division of Cardiac Surgery, Shisong Cardiac Centre, Kumbo, Cameroon; Radiology Unit, Douala General Hospital, Douala, Cameroon; Internal Medicine Service, Douala General Hospital, Douala, Cameroon

**Keywords:** aortic dissection, acute chest pain, hypertension, outcome, case report

## Abstract

Acute aortic dissection is the most frequent and deadly presentation of acute aortic syndromes. Its incidence is estimated at three to four cases per 100 000 persons per year. Its clinical presentation may be misleading, with misdiagnosis ranging between 14.1 and 38% in many series. A late diagnosis or absence of early and appropriate management is associated with mortality rates as high as 50 and 80% by the third day and second week, respectively, especially in proximal lesions. We report on the case of a 53-year-old man who presented with type A aortic dissection, misdiagnosed as acute myocardial infarction, who later died on day 12 of hospitalisation. Although a relatively rare condition, poor awareness in Africa probably accounted for the initial misdiagnosis. Thorough investigation of acute chest pain and initiation of clinical registries are potential avenues to curb related morbidity and mortality.

Cardiovascular diseases are the leading cause of death in the Western world and are on the rise in developing countries.^1^-^ 3^ Acute aortic syndromes include acute aortic dissection (AAD), intramural haematoma, penetrating aortic ulcer and ruptured thoracic aortic aneurysm.^3^,^4^ AAD is the most frequent and lethal presentation of acute aortic syndromes, with an incidence of three to four cases per 100 000 persons per year.^5^

There are several different classification systems of aortic dissection. The two most commonly used formats are the Debakey and Standford classifications, as shown in Table 1.

**Table 1:** Classification of acute aortic dissection

*Standford classification*		*DeBakey classification*		
*Type A*	*Type B*	*Type I*	*Type II*	*Type III*
Dissection involving the proximal aorta (ascending aorta, aortic arch) with or without extension to the descending aorta^7^,^8^	Dissection limited to the descending aorta^7^ (but may be extended to the abdominal segment)	Involving the ascending aorta and a variable amount of descending or thoracoabdominal aorta^7^	Dissection limited to the ascending aorta^8^	Dissection of the descending aorta either without (IIIa) or with (IIIb) involvement of the abdominal aorta^7^

In the absence of treatment, AAD type A has worse outcomes, with an initial mortality rate of 1% per hour, with 50 and 80% of the patients expected to die by the third day and second week, respectively. Progression of the dissection can be either anterograde or retrograde from the initial tear, with resultant malperfusion syndromes, acute coronary syndromes (ACS), cardiac tamponade or aortic valve insufficiency.^6^ Its clinical presentation may be misleading with misdiagnosis ranging from 14.1 to 38% seen in many series.^7^-^10^

The differential diagnosis of AAD may include acute coronary syndromes, pericarditis, pulmonary embolism, acute pancreatitis and peptic ulcer disease. AAD usually mimics ACS.^11^ Factors favouring misdiagnosis of AAD include clinical similarities with common diseases such as ACS, low regional epidemiology, and limited access to specific diagnostic imaging modalities in some regions. In one study, it was shown that the commonest factors favouring missed diagnosis of AAD were walk-in patients, anterior chest pain, severe or worst-ever pain and widened mediastinum, with walk-in mode of admission being the single strongest predictor of misdiagnosis.^8^

Diagnostic imaging studies are pivotal in confirming the diagnosis and classifying the extent of the dissection using either DeBakey (I, II and III) or Standford (A or B) classifications. AAD involving the ascending aorta (Standford type A) is a surgical emergency requiring swift repair of the aortic root or reconstruction of the ascending aorta and arch to improve prognosis, whereas dissections involving the descending aorta (Standford type B) are treated medically with the following surgical indications: propagation of the dissection, intractable pain or poor organ perfusion.^3^,^12^

## Case report

A 53-year-old sub-Saharan African man with poorly controlled hypertension was referred to the cardiac intensive care unit (CICU) by his cardiologist for the management of a sudden-onset, severe and intractable retrosternal chest pain of approximately 50 hours’ duration. The pain was tearing in character, radiating to the back and lumbar regions, non-positional and associated with shortness of breath and headache. 50 hours’ duration. The pain was tearing in character, radiating to the back and lumbar regions, non-positional and associated with shortness of breath and headache.

The electrocardiogram (ECG), done three hours after the onset of pain, showed sinus rhythm and non-specific repolarisation changes (flattened or inverted T waves in leads I, aVL and V3–V6). Although ECG changes were suggestive of left ventricular strain, the presence of chest pain and a mildly raised troponin level (0.11 μg/ml) favoured myocardial infarction, and the patient was started on low-molecular weight heparin (LMWH) at a therapeutic dose, aspirin and nitrates.

Persistence of the pain after initial therapy prompted referral to our centre. On examination, he was anxious, dyspnoeic (NYHA functional class III with a respiratory rate of 28 breaths/ min) and diaphoretic. His temperature was 36.9°C, heart rate was 79 beats/min, and blood pressure was 187/73 mmHg in the right arm and 145/56 mmHg in the left arm. Physical examination showed a systolic murmur (grade 3/6) in the aortic area, which radiated to the left carotid, but there were no signs of heart failure. The neurological examination was unremarkable.

**Fig. 1.**
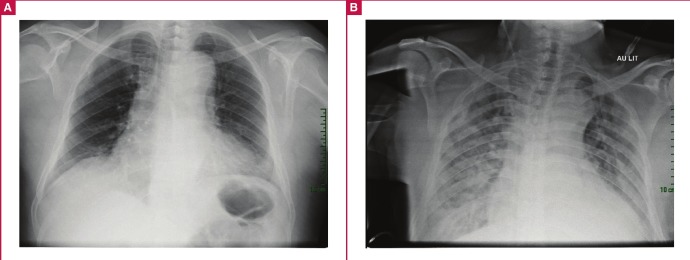
Anterior–posterior chest X-ray. A: At presentation showing enlargement of the mediastinum. B: On day 11 of hospitalisation showing bilateral interstitial heterogeneous opacities.

**Fig. 2:**
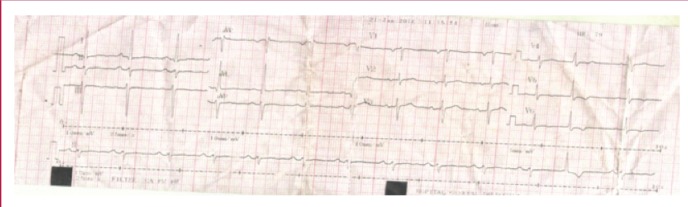
ECG at presentation showing non-specific ST-segment changes consistent with sub-epicardial ischaemia in the inferior and apico-lateral leads.

**Fig. 3:**
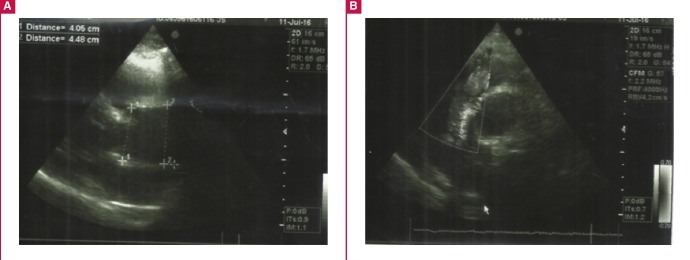
Echocardiography showing dilatation of the ascending aorta.

Chest X-ray (Fig. 1A) showed enlargement of the mediastinum with cuffing of the aortic knob. The ECG (Fig. 2) at our unit showed a normal sinus rhythm, normal QRS axis with sub-epicardial ischaemia in the inferior and apico-lateral leads. Echocardiography (Fig. 3) showed a dilated left atrium, good left ventricular systolic function (ejection fraction 72%), and severe aortic insufficiency with dilatation of the aortic root and ascending aorta (44 mm).

**Fig. 4:**
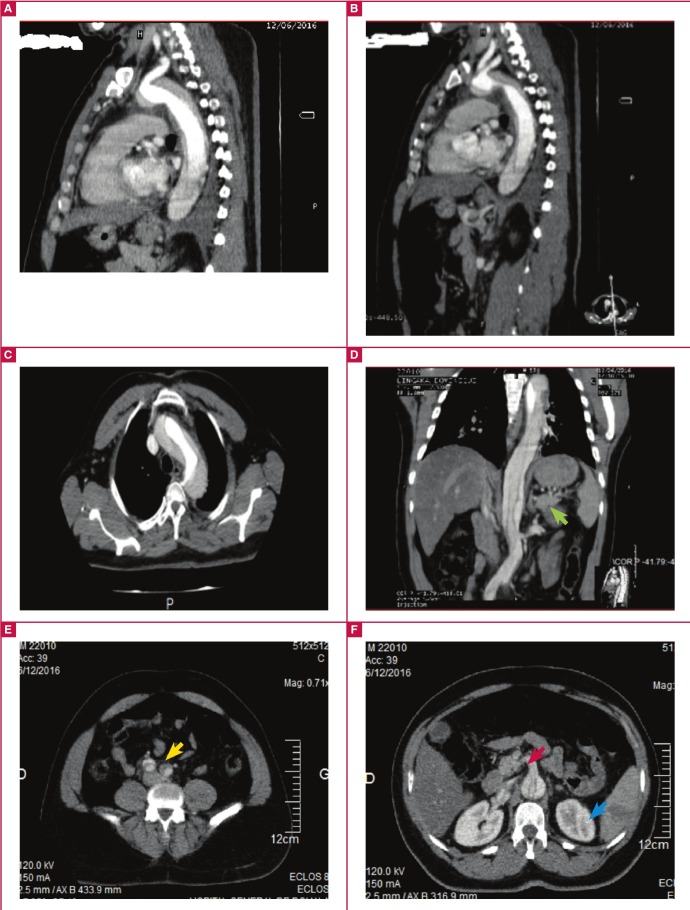
Contrast-enhanced CT angiogram of the thorax showing aortic dissection extending to the left renal (green arrow), iliac (yellow arrow) and superior mesenteric (red arrow) arteries and causing splenic infarction (blue arrow).

Contrast-enhanced CT (CECT) angiogram of the thorax (Fig. 4) showed dissection of the aorta from the ascending aorta to the iliac arteries, including the coeliac trunk and left renal artery, and causing splenic infarction. Doppler ultrasound of the carotid arteries did not show extension to the carotid arteries. These observations led to a working diagnosis of Standford type A acute aortic dissection. Table 2 shows biological investigations done at presentation and throughout hospitalisation.

**Table 2:** Serial biological investigations done at the emergency department and throughout hospitalisation

*Biological investigation*	*Presentation*	*Day 1*	*Day 4*	*Day 10*	*Day 11*
White cell count, × 106^6^ cells/l	6.8	9.5	7.3	5.2	17.7
C-reactive protein, mg/l	<6	7.21	30.72	310.43	ND
Haemoglobin, g/l	15.2	13.5	13.2	12.4	10.5
Serum creatinine, mg/l	17.2	12.3	ND	13.1	ND
Troponin I	2.26	0.69	ND	ND	0.15
Creatine kinase (CK), IU/l	200	ND	ND	ND	ND
CK-MB, IU/l	24.9	ND	ND	ND	ND
LDH, UI/l	455	ND	ND	ND	ND
D-dimers	24087	ND	ND	ND	ND
NT-pro BNP	117	ND	ND	ND	6,366

LDH = lactate dehydrogenase test; ND = not done

The patient was placed on high-flow oxygen at 5 l/min, nicardipine in an electric syringe titrated to a maximum of 10 mg/h, bisoprolol 5 mg/12 h, analgesics and compressive stockings. The LMWH was stopped. On day five of hospitalisation, he developed superficial thrombophlebitis on the left forearm (along the peripheral intravenous line). By day six of hospitalisation, blood pressure and heart rate targets (< 120/80 mmHg and < 60 beats/min, respectively) were achieved.

On day 10 of hospitalisation, the patient developed a temperature of 39.1°C and sudden dyspnoea at rest. Physical examination showed a heart rate of 119 beats/min, blood pressure of 124/76 mmHg and oxygen saturation of 98%. Chest examination revealed crepitation in both lung bases, more marked on the right. We decided on a presumptive diagnosis of severe pneumonia. A repeat chest X-ray (Fig. 1B) showed bilateral interstitial heterogeneous opacities.

The C-reactive protein (CRP) level was 310.43 mg/l with leucocytosis of 17.7 × 10^6^ cells/l (Table 2). Blood samples were collected for culture, and antibiotics (amoxicillin–clavulanic acid 1 g eight hourly and clarithromycin 1 000 mg 12 hourly) were introduced. Blood culture results (which returned after the patient’s demise) were positive for Klebsiella pneumonia. About three hours later he had persistent dyspnoea and hypoxaemia (SpO_2_ ≤ 65% and PaO_2_ ≤ 60 mmHg). He was intubated and during the process sustained a cardiac arrest. The patient later died on day 12 of hospitalisation following cardiopulmonary arrest despite life support.

## Discussion

AAD is characterised by separation of the layers of the aortic wall, resulting from the entry of extra-luminal blood through an intimal tear, producing a false lumen. Tears are commonly seen at areas of high stress, commonly in the anterior aortic wall just above the aortic valve (66%) and the posterior wall of the proximal descending aorta (33%). When blood enters through an intimal tear it passes longitudinally along the tunica media separating the intima from the adventitia.^13^ There are several different classification systems of aortic dissection. The two most commonly used formats are the DeBakey and Standford classifications, as described in literature.^12^,^14^

The typical presentation of AAD is a sudden, unexpected, intense retrosternal pain radiating to the back and/or abdomen, associated with asymmetrical blood pressure.^6^ Patients are typically hypertensive, middle aged or elderly and therefore the differential diagnosis would include acute myocardial infarction, acute coronary syndromes, pericarditis, pulmonary embolism, peptic ulcer disease and acute pancreatitis. Due to its possibility of extension to involve the mesenteric, iliac and renal arteries, other presentations may include intestinal ischaemia, stroke and renal failure.^5^ A misdiagnosis at presentation may occur in up to 38% of AADs, as well as being discovered during post mortem in 28% of cases without any prior identification or suspicion.^15^

Our patient presented with typical features of AAD, which was initially diagnosed as myocardial infarction (MI), probably due to the relative rarity of the condition compared to MI in our setting. However, a thorough clinical assessment and high index of suspicion may have picked up suggestive clinical features. Furthermore, a chest X-ray, which usually shows enlargement of the mediastinum with knobbing of the aorta in about 60% of the cases was not done.^16^ This further emphasises the importance of a chest X-ray among first-line investigations in the management of acute chest pain.

According to the American Heart Association 2010 guidelines for the management of acute thoracic disease, possible ECG findings in the evaluation of AAD include: 30% normal ECG, 40% non-specific ST-segment changes, 26% left ventricular hypertrophy and 15% signs of ischaemia.^15^ Our patient had non-specific ST-segment changes consistent with myocardial ischaemia. CECT angiogram of the thorax was used to confirm the diagnosis of Standford type A AAD in the indexed case, with the dissection extending to the iliac, mesenteric and left renal arteries.

Although the diagnosis of AAD type A was made relatively late in our patient, he was not operated on because of lack of local cardiosurgical centres, financial constraints and his refusal of evacuation to another country. The target blood pressure and heart rate, as described in the literature,^3^ were achieved after six days of hospitalisation. Potential contributing factors to the fatality included late referral and diagnosis, initial treatment with LMWH at therapeutic dose and aspirin, lack of local cardiosurgical centres for emergency surgery, and severe sepsis.

## Conclusion

Despite the relative rarity of AAD in sub-Saharan African settings, this case highlights the importance of thorough early clinical assessment and investigation in the emergency room of patients with acute chest pain. Furthermore, limited resources common in low-income settings contribute to this health burden. The initiation of clinical registries is a potential avenue to increase awareness around these fatal conditions and thereby contribute to reduction of cardiovascular-related morbidity and mortality.
